# Why we need to abandon fixed cutoffs for goodness-of-fit indices: An extensive simulation and possible solutions

**DOI:** 10.3758/s13428-023-02193-3

**Published:** 2023-08-28

**Authors:** Katharina Groskurth, Matthias Bluemke, Clemens M. Lechner

**Affiliations:** 1 GESIS – Leibniz Institute for the Social Sciences, Mannheim, Germany; 2 University of Mannheim, Graduate School of Economic and Social Sciences, Mannheim, Germany; 3 Technical University of Darmstadt, Darmstadt, Germany

**Keywords:** Goodness-of-fit, Fit index, Ordered categorical data, Confirmatory factor analysis, Structural equation modeling

## Abstract

To evaluate model fit in confirmatory factor analysis, researchers compare goodness-of-fit indices (GOFs) against fixed cutoff values (e.g., CFI > .950) derived from simulation studies. Methodologists have cautioned that cutoffs for GOFs are only valid for settings similar to the simulation scenarios from which cutoffs originated. Despite these warnings, fixed cutoffs for popular GOFs (i.e., χ^2^, χ^2^/*df*, CFI, RMSEA, SRMR) continue to be widely used in applied research. We (1) argue that the practice of using fixed cutoffs needs to be abandoned and (2) review time-honored and emerging alternatives to fixed cutoffs. We first present the most in-depth simulation study to date on the sensitivity of GOFs to model misspecification (i.e., misspecified factor dimensionality and unmodeled cross-loadings) and their susceptibility to further data and analysis characteristics (i.e., estimator, number of indicators, number and distribution of response options, loading magnitude, sample size, and factor correlation). We included all characteristics identified as influential in previous studies. Our simulation enabled us to replicate well-known influences on GOFs and establish hitherto unknown or underappreciated ones. In particular, the magnitude of the factor correlation turned out to moderate the effects of several characteristics on GOFs. Second, to address these problems, we discuss several strategies for assessing model fit that take the dependency of GOFs on the modeling context into account. We highlight tailored (or “dynamic”) cutoffs as a way forward. We provide convenient tables with scenario-specific cutoffs as well as regression formulae to predict cutoffs tailored to the empirical setting of interest.

In social and behavioral science research, researchers commonly employ goodness-of-fit indices (GOFs) to evaluate the fit of latent variable models such as confirmatory factor analysis (CFA) models. The most widely used GOFs (e.g., Jackson et al., 2009) are the chi-square test statistic divided by the model degrees of freedom (χ^2^/*df*), the comparative fit index (CFI), the root mean square error of approximation (RMSEA), and the standardized root mean square residual (SRMR). In addition, researchers often rely on the traditional chi-square test statistic of exact model fit (χ^2^). Although strictly speaking not a GOF but a formal test, researchers use χ^2^ in much the same way as they use GOFs (see also Jöreskog & Sörbom, 1993), which is why we henceforth simply subsume it under the same rubric.

Cutoffs for GOFs, on which researchers’ binary decisions about accepting or rejecting a model rest, are based on simulation studies. Simulation studies represent highly controlled situations in which—different from the analysis of real data—researchers know and have control over the population (or data-generating) model. Having specified a data-generating population model, researchers determine (the size of) model misspecification in the analysis model. Then, they observe how GOFs respond to such misspecification by simulating many datasets from the population model and fitting an analysis model to each dataset. Based on the distribution of the resulting GOFs, researchers derive cutoffs for these GOFs so that a critical level of misspecification leads to model rejection. What constitutes a “critical” level of misspecification and yields a reasonable cutoff is an arbitrary decision (e.g., deeming a Type I error rate of 5% for a χ^2^-based decision “acceptable”).

In the past two decades, Hu and Bentler’s (1999) cutoffs have been the most prominent and widely used ones. At the time of this writing, their article boasts more than 95,000 citations on Google Scholar, making it one of the most highly cited articles across all social and behavioral sciences. According to these authors, CFI ≥ .950, RMSEA ≤ .060, and SRMR ≤ .080 point to good model fit. More recently, Reußner (2019) and Rutkowski and Svetina (2014) proposed similar cutoffs. Bollen (1989) outlined that the observed χ^2^ value should not exceed a critical χ^2^ value, which varies with the model degrees of freedom and is based on statistical principles rather than derived from simulation studies (see Moshagen & Erdfelder, 2016, for additional thoughts about critical values). Ullman (2014) suggested that a ratio of χ^2^/*df* below 2 indicates an empirically well-fitting model.

However, there are severe problems with relying on any of these fixed cutoffs for GOFs in model evaluation (e.g., McNeish & Wolf, 2023a, b). The key underlying issue is that simulation studies can only cover a limited set of scenarios (i.e., combinations of data and analysis characteristics). These scenarios are far from covering all possible combinations of data (e.g., distribution of response options) and analysis characteristics (e.g., the number of factors and the estimator) that researchers will encounter in applied settings. If GOFs reacted solely to model misspecification predictably and uniformly, the confines of simulation studies would not pose a major problem. If, by contrast, GOFs reacted not only to misspecification but also to other characteristics of the data and analysis, their validity for judging the model fit might be severely compromised. We henceforth refer to the undesirable dependence of GOFs on data and analysis characteristics (other than the model misspecification one seeks to detect) as *susceptibility*. We contrast it with the desirable *sensitivity* of GOFs to misspecification.

The susceptibility to data and analysis characteristics of GOFs is not a hypothetical concern. Although GOFs were designed to detect and quantify (degrees of) model misspecification and to ideally not depend on any data or analysis characteristic (Schermelleh-Engel et al., 2003), they apparently do, as identified in several studies (for an overview, see Niemand & Mai, 2018). Therefore, established cutoffs for GOFs are valid only in empirical settings (i.e., combinations of data and analysis characteristics) that closely resemble the scenarios covered by the simulations from which these cutoffs were derived. The range of scenarios covered by existing simulations is dwarfed by the diversity and complexity of empirical settings encountered in research applications. For example, in their seminal paper that led to the now canonical cutoffs for GOFs, Hu and Bentler (1999) simulated data from a three-factor model with five indicators per factor. They fit models to these data that were either correctly specified or misspecified (either by omitting cross-loadings or omitting factor covariances). Although these population models were consistent with their goal to study the sensitivity of GOFs to misspecification and the susceptibility of GOFs to other characteristics, it is obvious that their findings cannot be easily generalized to one-factor models for which omitted cross-loadings or factor covariances do not even exist (see also McNeish & Wolf, 2023b). As this example illustrates, cutoffs for GOFs may lack external validity—applying the same set of cutoffs indifferently to many different empirical settings may mislead researchers and promote erroneous inferences about model fit and substantive questions attached to the model.

Unfortunately, current reporting practice shows that researchers apply cutoffs rather uniformly, even in the presence of data or analysis characteristics that can differ markedly from the ones in the simulation studies (for an overview, see Jackson et al., 2009; McNeish & Wolf, 2023a). It appears that repeatedly voiced concerns against overgeneralizations of cutoffs have gone largely unheeded (e.g., Heene et al., 2011; Markland, 2007; Marsh et al., 2004; McNeish & Wolf, 2023a; Niemand & Mai, 2018; Nye & Drasgow, 2011). The widespread—in fact, near-universal—practice of relying on (fixed) cutoffs for GOFs in model evaluation is alarming, given the lingering uncertainty about the applicability of fixed cutoffs for GOFs to scenarios hitherto uncharted by simulation studies.

Just *how* problematic is the practice of using fixed cutoffs for GOFs? And *what* alternatives to fixed cutoffs can researchers use? We surmise that a lack of awareness both about the serious problems with fixed cutoffs and about the availability of alternative approaches contributes to the abiding use of fixed cutoffs. In our study, we therefore review extant evidence on the susceptibilities of GOFs to data and analysis characteristics. We then present an extensive simulation study that integrates, replicates, and extends previous simulation studies and represents the most in-depth simulation on the sensitivity and susceptibility of GOFs to date. This simulation reinforces the conclusion that cutoffs cannot be easily generalized to arbitrary analytical scenarios, such that fixed cutoffs are likely invalid in most situations. We then review several time-honored and promising emerging alternatives for model fit evaluation that do not rely on fixed cutoffs. We argue that cutoffs must be tailored to the empirical setting of interest. Based on the large-scale simulation study, we generated user-friendly tables with scenario-specific cutoffs and developed regression formulae to predict cutoffs for an empirical setting of interest.

## Susceptibilities of GOFs to data and analysis characteristics: A review of previous findings

GOFs are intended to enable evaluations of model fit, specifically, to help detect non-negligible model misspecification.^1^ However, as previous investigations have shown, GOFs are susceptible to a multitude of data and analysis characteristics other than the model misspecification they are meant to detect (e.g., Beauducel & Herzberg, 2006).^2^ These characteristics include the sample size (e.g., DiStefano et al., 2019), type of estimator (e.g., Xia & Yang, 2019), the number of indicators^3^ (e.g., Kenny & McCoach, 2003), number and distribution of response options (e.g., Xia & Yang, 2018), the magnitude of factor loadings (e.g., Heene et al., 2011), and the factor correlation (e.g., Beauducel & Wittmann, 2005).

Moreover, the impact of these characteristics on GOFs differs between correctly specified and misspecified models—which we review in detail here. For correctly specified models, GOFs (i.e., χ^2^, χ^2^/*df*, CFI, RMSEA, and SRMR) typically signaled better model fit with increasing sample size (e.g., Beauducel & Herzberg, 2006; Chen et al., 2008; DiStefano et al., 2019; Kenny et al., 2015; Sharma et al., 2005; Shi et al., 2019). Likewise, GOFs (i.e., CFI and SRMR) of correctly specified models pointed to better fit with a higher magnitude of factor loadings (and a lower magnitude of residual variances; Beierl et al., 2018; Heene et al., 2011; Shi et al., 2019). GOFs (i.e., CFI, RMSEA, and SRMR) also signaled better model fit with symmetric instead of asymmetric response distributions (Reußner, 2019). The influence of the number of indicators on GOFs of correctly specified models interacted with the sample size: At small sample sizes (e.g., *N* = 100), GOFs (i.e., χ^2^/*df*, CFI, and RMSEA) indicated worse model fit when indicators of similar psychometric quality were added (Kenny & McCoach, 2003; see also Sharma et al., 2005; Shi et al., 2019). At large sample sizes (*N = *1000), GOFs (i.e., χ^2^/*df* and RMSEA) pointed to better model fit as the number of indicators increased (only CFI was no longer affected; Kenny & McCoach, 2003). Per statistical definition, χ^2^ increases when adding indicators without further restrictions to the model (Bollen, 1989). Only the magnitude of factor covariance/correlation in correctly specified multidimensional models seemed to be a model characteristic to which GOFs (i.e., χ^2^, CFI, RMSEA, and SRMR) are impervious (Beauducel & Herzberg, 2006; Beierl et al., 2018).

For misspecified models, studies found that GOFs (i.e., χ^2^, χ^2^/*df*, CFI, and SRMR^4^) typically signaled worse model fit with an increasing number of indicators (yet vice versa for RMSEA, e.g., DiStefano et al., 2019; Kenny & McCoach, 2003; Savalei, 2012; Shi & Maydeu-Olivares, 2020; Shi et al., 2019). Likewise, GOFs (i.e., χ^2^, RMSEA, and SRMR) of misspecified models showed worse model fit with a higher magnitude of factor loadings (and a lower magnitude of residual variances)—CFI reacted inconsistently across studies (Beierl et al., 2018; Hancock & Mueller, 2011; Heene et al., 2011; McNeish et al., 2018; Shi et al., 2019; Shi & Maydeu-Olivares, 2020; Shi et al., 2018b; cf. Moshagen & Auerswald, 2018, who kept the degree of misspecification and residual error variances constant). GOFs of misspecified models also signaled worse model fit when the response distribution was symmetric compared to asymmetric distributions (Reußner, 2019; Xia & Yang, 2018).^5^ Similarly, GOFs (i.e., χ^2^ and SRMR) of models with uncorrelated factors pointed to worse fit than with correlated factors for specific misspecification (i.e., with unmodeled cross-loadings that all have the same sign; Beauducel & Wittmann, 2005). The influence of the sample size on GOFs of misspecified models was mixed: χ^2^, χ^2^/*df*, and RMSEA indicated worse model fit with increasing sample size, whereas CFI and SRMR suggested better model fit (e.g., Beauducel & Wittmann, 2005; DiStefano et al., 2019; Nye & Drasgow, 2011).

GOFs also depended directly on the estimator used. Researchers frequently apply maximum likelihood (ML; Bollen, 1989) or its robust cousin MLR that corrects the χ^2^ test statistic and standard errors of ML-estimated parameters for non-normality (Muthén & Muthén, 1998-2017; Yuan & Bentler, 2000). Both estimate parameters based on unstandardized covariances or Pearson correlations. Diagonally weighted least squares (DWLS) based on polychoric correlations or the corresponding mean- and variance-adjusted (WLSMV) χ^2^ test statistic (and standard errors) are less common (Muthén, 1984; Muthén et al., 1997). However, WLS estimators are gaining relevance as more and more researchers note their utility and suitability for analyzing ordered-categorical data, such as data from rating scales with few response options only (for an overview of the estimation procedures, see Li, 2016). In simulations, the DWLS-/WLSMV-based GOFs (i.e., χ^2^, CFI, and RMSEA) generally pointed to better model fit than ML-based ones (Beauducel & Herzberg, 2006; Nye & Drasgow, 2011; Xia & Yang, 2019) for correctly specified and misspecified models.^6^ For SRMR, the effect was reversed for correctly specified models; it indicated worse fit with DWLS than ML (Beauducel & Herzberg, 2006). The type of estimator also moderated other influences: DWLS/WLSMV-based GOFs (i.e., χ^2^, χ^2^/*df*, CFI, and RMSEA) generally suggested worse fit with a higher (compared to a lower) number of response options in both correctly specified and misspecified models (Beauducel & Herzberg, 2006; Xia & Yang, 2018).

In sum, previous simulation studies provide ample evidence that GOFs are susceptible to extraneous influences (other than misspecification). Moreover, GOFs sometimes behave differently in correctly specified compared to misspecified models, and different data and analysis characteristics may interact in complex and unforeseen ways. However, no prior simulation study has investigated *all* aforementioned influences on GOFs in conjunction. Instead, most researchers focused on one or two characteristics thought to impact GOFs. For instance, research has repeatedly investigated the effects of different magnitudes of factor loadings on GOFs (e.g., Beierl et al., 2018; Heene et al., 2011; Shi et al., 2019). Research has also often investigated the effects of the number of response options and type of estimator on GOFs in tandem (e.g., Xia & Yang, 2019).

Because no prior simulation study has investigated all the aforementioned influences on GOFs in conjunction, it is not fully clear how susceptible GOFs are to the joint influences of these characteristics. The aforementioned characteristics may influence GOFs not only in the form of main effects but also through interactions (e.g., sample size × number of response options), which may attenuate or aggravate any known biases of GOFs. Further, it remains unknown which influences on GOFs, identified by prior simulations, would replicate when several data and analysis characteristics are considered jointly. Only an extensive simulation that jointly considers characteristics known to influence GOFs can provide such relevant insights: First, it integrates findings from previous studies and, thus, produces comprehensive, hitherto uninvestigated simulation scenarios. Second, it can replicate and, thus, solidify previously known patterns. Third, it can identify previously unknown patterns (e.g., interaction effects) and, thus, reveal the characteristics’ complex interplay. Thereby, the simulation study provides a more complete picture of the performance of GOFs in different scenarios. Although such studies soon reach a high level of complexity, replication-extension studies such as our extensive simulation study are highly relevant and offer unique advantages for cumulative science (Bonett, 2012). Through the generalization of effect sizes across contexts (i.e., simulation scenarios across studies), replication checks, and the investigation of moderation effects (i.e., interaction terms), replication-extension studies can expose misleading findings and too narrow inferences in earlier studies. This inspired us to carry out the extensive simulation study that we present in the following sections.

## The present simulation

### Aims of the simulation

We conducted a Monte Carlo simulation study (for more details on Monte Carlo simulations, see Boomsma, 2013) to integrate, replicate, and extend findings from previous simulations on GOFs. Focusing on CFA models, we investigated the joint impact of a wide range of data and analysis characteristics on GOFs. CFA models are among the most widely used latent variable models and form the basis for a wide range of applications, such as evaluating measurement instruments, testing structural theories of psychological constructs, and studying the relations among constructs.

### Design of the simulation

To ensure its external validity, we designed our simulation to cover realistic scenarios typically encountered in social and behavioral science research. Each scenario comprised a population model with a different combination of characteristics. For each scenario, we drew 1000 random samples of varying size based on that population model. Additionally, we incorporated a correctly specified or misspecified analysis model that we fit to each randomly sampled dataset employing different estimators.

**Fig. 1:**
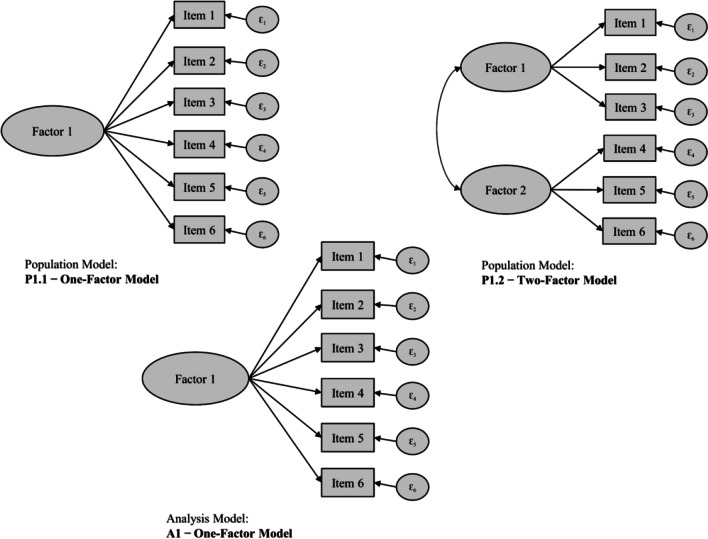
Exemplary population and analysis models for the factor dimensionality scenario. *Note.* Models with six indicators for exemplary purposes

We considered different combinations of data-generating (i.e., population) and analysis models to cover a breadth of constellations that may occur in real-world settings. In the first combination, the population model was either a one-factor or correlated two-factor model. We fit a one-factor analysis model to data generated from both population models (i.e., one-factor and correlated two-factor models). Consequently, each analysis model was either correctly specified or misspecified relative to the population model regarding factor dimensionality. We varied the factor correlations in the two-factor population model (*r* = .70, .50, or .30) to induce different sizes of misspecification in the one-factor analysis model. Thus, the misspecification induced by the different factor correlations in the population model (*r* = .70, .50, or .30) corresponded to a parameter difference of .30, .50, or .70, when viewed from the perspective of perfectly correlating factors (*r* = 1, representing essentially a single factor) in the analysis model. Figure 1 shows exemplary population and analysis models for these so-called factor dimensionality scenarios.

**Fig. 2:**
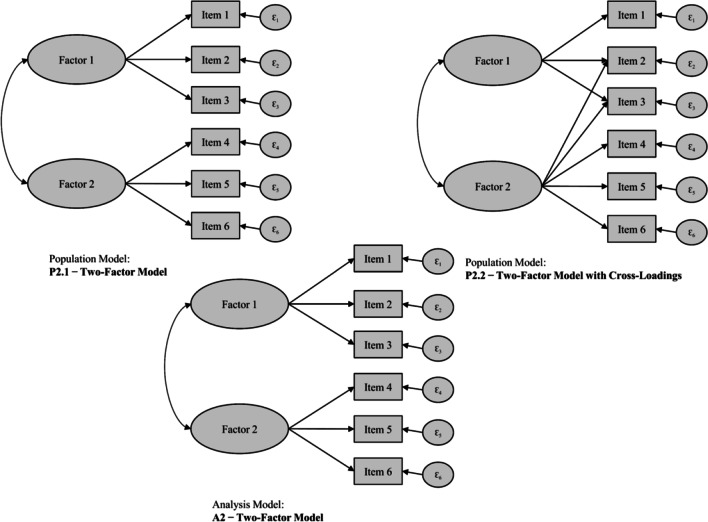
Exemplary population and analysis models for the cross-loading scenarios*. Note.* Models with six indicators, correlated factors, and two cross-loadings (i.e., cross-loadings exist for 33% of all six indicators) for exemplary purposes

As a second combination, the two-factor population model either did not or did include cross-loadings on one factor. We consistently fit a two-factor analysis model without any cross-loadings to data generated from both types of population models (i.e., without and with cross-loadings). Thus, each analysis model was either correctly specified or misspecified regarding the presence and magnitude of unmodeled cross-loadings. We stipulated that either 17% or 33% of all indicators had cross-loadings. The cross-loadings had a standardized loading magnitude of either .20 or .30. This resulted in different proportions and magnitudes of model misspecification in the analysis models in which these cross-loadings went unmodeled. Figure 2 shows exemplary population and analysis models for these so-called cross-loading scenarios.

In both combinations, we varied a total of six different data and analysis characteristics to which GOFs may be susceptible according to previous research: type of estimator, number of indicators, number of response options, distribution of response options, loading magnitude, and sample size.^7^ With either correctly specified or misspecified two-factor analysis models regarding cross-loadings in the population model, we additionally varied the factor correlation (i.e., factors were either correlated or uncorrelated). The two factors of the population and analysis models were either allowed to correlate or forced to be uncorrelated. With either correctly specified or misspecified one-factor analysis models regarding factor dimensionality in the population model, we cannot vary the factor correlation in either population or analysis models. Thus, we only varied the factor correlation in a subset of scenarios (i.e., the cross-loading scenarios). Table 1 summarizes the different scenarios analyzed in this study—which were oriented upon typical settings encountered in empirical research.

**Table 1:** Simulation scenarios

	Realization For all population models: Factor variances = 1, Indicator variances = 1 Residual variances = $1 -\left(Var(F1\right)\times {\lambda }_{F1}^{2}+ Var(F2)\times {\lambda }_{F2}^{2}+2\times {\lambda }_{F1}\times {\lambda }_{F2}\times Cov\left(F1, F2\right))$ Replications = 1000				
Characteristic	(1)		(2)		Literature on typical settings used for operationalization
	Factor dimensionality		Cross-loadings		
Population model	One-factor model	Two-factor model	Two-factor model	Two-factor model with cross-loadings	
Analysis model	One-factor model	One-factor model	Two-factor model	Two-factor model without cross-loadings	
Specification	Correct	Misspecified	Correct	Misspecified	
Magnitude of misspecification	0	.30, .50, .70	0	.20, .30	
Proportion of misspecification	0	1	0	.17, .33	
Estimator	ML,	ML,	ML,	ML,	
	MLR^c^,	MLR^c^,	MLR^c^,	MLR^c^,	
	DWLS,	DWLS,	DWLS,	DWLS,	
	WLSMV	WLSMV	WLSMV	WLSMV	
Number of indicators	6, 12	6, 12	6, 12	6, 12	Rammstedt & Beierlein (2014)
Response options	3, 5, 7	3, 5, 7	3, 5, 7	3, 5, 7	Clark & Watson (2019); Simms et al. (2019)
Distribution of responses^a^	Symmetric	Symmetric	Symmetric	Symmetric	Blanca et al. (2013)
	(skew=0.00),	(skew=0.00),	(skew=0.00),	(skew=0.00),	
	Asymmetric	Asymmetric	Asymmetric	Asymmetric	
	(skew=0.65)	(skew=0.65)	(skew=0.65)	(skew=0.65)	
Loading magnitude	.40, .60, .80	.40, .60, .80	.40, .60, .80	.40, .60, .80	Soto & John (2017)
Sample size	200, 500, 2000	200, 500, 2000	200, 500, 2000	200, 500, 2000	Bilsky et al. (2011); Comrey & Lee (1992); Nießen et al. (2019)
Factor correlation	$NA$	$NA$	.00, .30	.00, .30	Groskurth et al. (2021); Kim et al. (2022); Lee & Cagle (2017); Soto & John (2017)
			(factors not allowed vs. allowed to correlate)	(factors not allowed vs. allowed to correlate)	
Total number of scenarios	432	1296	864	3456	
	(*n* = 432,000)	(*n* = 648,000)	(*n* = 864,000)	(*n* = 3,456,000)	
	1728		4320		
	(*n* = 1,728,000)		(*n* = 4,320,000)		
	6048				
	(*N* = 6,048,000)				
Resampled data^b^	7%				
Non-convergence	2%				
	(Final *N* = 5,956,844)				

*F1 *first factor, *F2 *second factor, *Var *variance, λ factor loading, *Cov *covariance, *N *total number of datasets, *n *subset of datasets, *NA* not applicable (i.e., scenario not tested or testable). ^a^ For all scenarios: Excess kurtosis ≈ −0.80. ^b^ We had to re-simulate data whenever cell frequencies for any response option of any indicator resulted in fewer than five data points, because DWLS/WLSMV can only estimate thresholds for response options that do contain observations. ^c^ Yuan and Bentler (2000) corrected χ^2^ test statistic

In our simulation study (see Table 1), all factors in the population models were normally distributed latent variables with unit variance. Observed indicators were also normally distributed with unit variance. Residual variances varied based on the population model parameters (i.e., loadings and, if applicable, factor correlation). We identified all analysis models by fixing the loading of the first indicator of each factor to unity. Unlike ML estimation, DWLS (and, accordingly, also WLSMV) include thresholds in the model parameterization that pertain to intermediate continuous latent response variables, which translate the use of response options depending on the standing of the latent response variable but also require identification themselves. To identify DWLS/WLSMV-based analysis models, we followed Millsap’s (2011) procedure in line with the theta parameterization. Unlike delta parameterization, which fixes the residual variances of the latent response variables to one, theta parameterization scales the distribution of latent response variables by fixing their variances to one (Muthén & Asparouhov, 2002).

We considered the following GOFs: χ^2^ (Bollen, 1989) χ^2^/*df*, CFI (Bentler, 1990; see also Widaman & Thompson, 2003), RMSEA (Steiger, 1990; see also Chen, 2007), and SRMR (Bentler, 1995; Hu & Bentler, 1999). Generally, GOF values closer to zero point to bad fit, except for CFI where values closer to one point to good fit. For interested readers, we included the computational details of GOFs in Additional File 1 of the Supplementary Online Material.

The final analysis contained GOFs for *N* = 5,956,844 models that converged (non-converged models were culled). We used R 3.6.3 (R Core Team, 2020) for all analyses. All R packages we used are documented in our R code. Two packages were particularly central to our analyses: We generated data with MASS 7.3-53 (Venables & Ripley, 2002) and fit the analysis models to the data with lavaan 0.6-7 (Rosseel, 2012). We took all GOFs from the lavaan output except for the script-based computation of χ^2^/*df*. For complete reproducibility, we monitored the R package versions via renv 0.12.2 (Ushey, 2020) and set random number generator seeds for the R code. We did not preregister the design and analysis of this non-empirical study. The full code is available on the Open Science Framework (OSF) at https://osf.io/e6kxa/?view_only=946034c00dee431897f67ca7ded58918.

### Statistical analyses

The outcomes of interest were the *sensitivity* of GOFs to model misspecification and their *susceptibility* to influences other than model misspecification, such as the type of estimator or sample size. We analyzed the sensitivity and susceptibility via descriptive and inferential statistics along four main steps. First and foremost, we inspected the distributions of GOFs across the different scenarios. Second, we looked at zero-order correlations between the GOFs and simulation characteristics to get a first impression of their sensitivity and susceptibility. Third, we examined the characteristics’ main and interaction effects on GOFs, including linear and quadratic terms, in multivariate regression.

The multivariate regression included two-way but not any higher-order interactions, for three reasons: First, technical restrictions prevented analyzing higher-level interactions. The biglm function from the biglm package in R (Lumley, 2013) was designed to handle big data. However, the biglm function limits the number of independent variables, thereby restricting the number of interaction effects in complex models. A second, more substantive reason was that the purpose of running regression models was to solidify, from a multivariate perspective, and quantify the various influences on GOFs that simpler analysis (e.g., descriptive statistics) might suggest. Two-way interactions already suffice to demonstrate whether GOFs are subject to joint (and potentially more complex) influences of various characteristics. Yet another reason why we focused on two-way interactions is to preserve straightforward interpretability and keep the exposition simple. Whereas two-way interactions are readily interpretable, three- or even four-way interactions would complicate matters beyond a point where they add much value.

Finally, we visually inspected selected major influences on GOFs. We selected those characteristics for visualization which appeared to have a large effect (or complex impact) on GOFs in the preceding analyses. The visualization permits an in-depth interpretation while compensating for the lack of higher-order interaction effects in the regression model.

## Simulation results

### Sensitivity of GOFs: Descriptive statistics

**Fig. 3:**
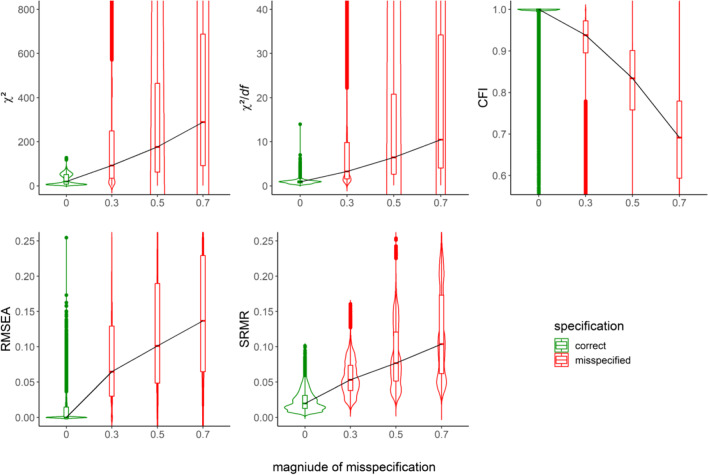
Distribution of GOFs for scenarios with correctly specified and misspecified factor dimensionality through the manipulation of the factor correlation. *Note*. GOFs in their original metric and direction. *Y*-axis restricted to improve readability

**Fig. 4:**
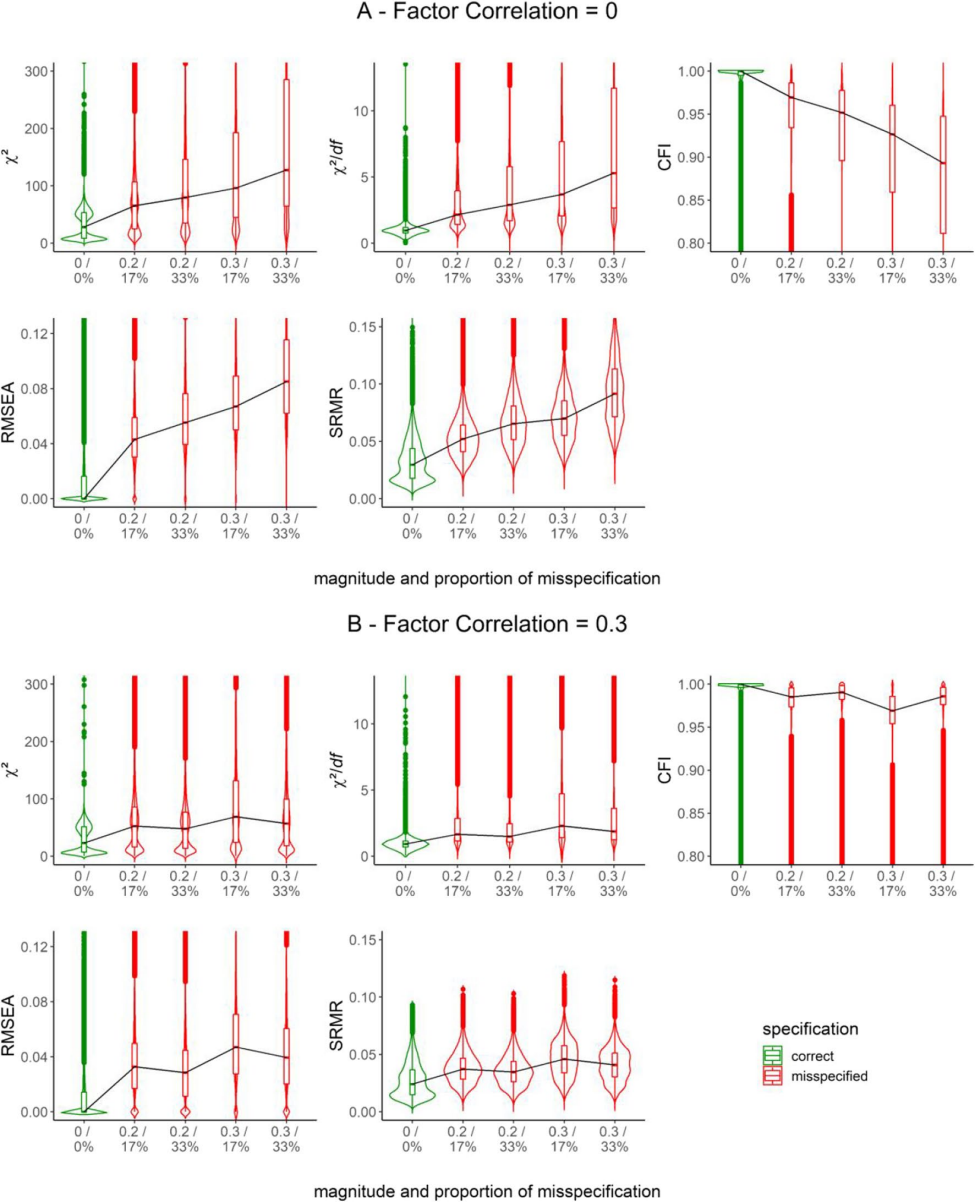
Distribution of GOFs for scenarios with correctly specified and misspecified models through the manipulation of cross-loadings. *Note*. *X*-axis levels refer to the magnitude of misspecification and proportion of misspecification, separated by a slash. GOFs in their original metric and direction. *Y*-axis restricted to improve readability

We first inspected how GOFs (i.e., χ^2^, χ^2^/*df*, CFI, RMSEA, and SRMR) were distributed across correctly specified and misspecified models in different scenarios (pooled across all the other relevant simulation characteristics). Figure 3 shows these distributions as violin plots for either correctly specified or misspecified models regarding factor dimensionality (i.e., one-factor analysis models for either a one-factor or two-factor population model). Similarly, Figure 4 shows distributions by the magnitude and proportion of cross-loadings in the population model that went unmodeled in the analysis model (i.e., two-factor analysis model for two-factor population model either without or with cross-loadings). We further split Fig. 4 into uncorrelated and correlated factor scenarios (factor correlation = .00 or .30) shown in Panels A and B, respectively. In Figs. 3 and 4, the *Y*-axis represents the relevant range of values for each GOF in its original metric and direction. The *X*-axis represents different degrees of severity of the misspecification, with the correctly specified model as a point of reference shown in green. The black trace line horizontally connects the GOF medians from different scenarios to reflect trends. Tables A1 and A2 in Additional File 2 of the Supplementary Online Material provide detailed descriptive statistics.

As expected, all GOFs signaled worse model fit with increasing *magnitudes* of misspecification in Figs. 3 and 4, evidenced by medians shifting toward unfavorable fit values. That is, all GOFs detected the misspecification of factor dimensionality and the misspecification due to increasingly unmodeled cross-loadings.

However, in Fig. 4, there are distinct influences of the *proportion* of unmodeled cross-loadings on GOFs in uncorrelated and correlated factor scenarios. For uncorrelated factors, an increasing proportion of misspecification shifted the GOF distribution toward more unfavorable values. For correlated factors, higher proportions of unmodeled cross-loadings resulted in lower medians of each GOF distribution (as the zigzag trace line indicates). Consequently, as the number of indicators with unmodeled cross-loadings increased, GOFs tended to indicate better, not worse, model fit. We did not expect that pattern and, thus, attend to it in more detail in the Discussion.

### Sensitivity and susceptibility of GOFs: Bivariate associations with characteristics

Next, we quantified how GOFs responded to the different characteristics in correctly specified and misspecified models. We computed Kendall’s tau-b to measure the bivariate association between each simulation characteristic and GOF to get a first impression of their sensitivity and susceptibility. The bivariate analysis revealed that GOFs were sensitive to the magnitude of misspecification, as expected, but less so (or even not at all) sensitive to the proportion of misspecification (the latter when cross-loadings remained unmodeled). GOFs were also strongly susceptible to (i.e., associated with) several extraneous characteristics in correctly specified and misspecified models, especially the type of estimator, loading magnitude, sample size, and factor correlation. In misspecified models, correlations of GOFs with data and analysis characteristics were often larger than those with misspecification (especially with loading magnitude and factor correlation)—implying that misspecification remains hidden in certain scenarios. For space reasons, the bivariate analysis is not included here but in Additional File 3 of the Supplementary Online Material.

### Sensitivity and susceptibility of GOFs: Multivariate analysis with joint effects of characteristics

Then, for all GOFs, we examined the joint effects of the characteristics combined, including their two-way interaction effects, in a regression analysis using a least squares estimator (Lumley, 2013; Miller, 1992). We modeled quadratic effects in addition to linear ones for independent variables with more than two levels. Table 2 summarizes the detailed regression results portrayed in Table A3 (for correctly specified models) and Table A4 (for misspecified models) in Additional File 4 of the Supplementary Online Material in terms of the direction of effects (not actual results or effect sizes). Table A5 in Additional File 4 of the Supplementary Online Material compares the findings from Table 2 with ones already identified in the literature review. This comparison suggested that we (1) replicated and, thus, solidified several known influences, (2) extended research by identifying hitherto unknown or underappreciated influences, and (3) extended research by characteristics that were not as influential as previously assumed when jointly considering multiple characteristics in a multivariate analysis.

**Table 2:** Summary of the sensitivities and susceptibilities of GOFs to model misspecification and other influences

Independent variables	Dependent variables									
	χ^2^		χ^2^/*df*		CFI		RMSEA		SRMR	
	Correct (1F/2F)	Misspecified (Dim./Load.)	Correct (1F/2F)	Misspecified (Dim./Load.)	Correct (1F/2F)	Misspecified (Dim./Load.)	Correct (1F/2F)	Misspecified (Dim./Load.)	Correct (1F/2F)	Misspecified (Dim./Load.)
	Main effects									
Misspecification magnitude	*NA*	−	*NA*	−	*NA*	−	*NA*	−	*NA*	−
Misspecification proportion^a^	*NA*	−	*NA*	−	*NA*	−	*NA*	−	*NA*	−
Estimator (Reference ML)										
MLR		− (Dim.)		− (Dim.)	+ (2F)			− (Dim.)	*NA*	*NA*
DWLS	+	− (Load.)	+	− (Load.)	−	+ / −	+ (1F)	− (Load.)	− (2F)	− (Load.)
WLSMV		− (Load.)		− (Load.)		− (Load.)		− (Load.)	*NA*	*NA*
Number of indicators	−									
Response options										
Asymmetric (Reference symmetric)	−		−				− (1F)			
Loading magnitude		−		−		+ (Load.)		−		− (Dim.)
Sample size									+	
Correlated factors (.30, Reference .00)^a^		+		+		+		+		+
	Large two-way interaction effects									
Misspecification magnitude ×										
DWLS	*NA*	− (Load.)	*NA*	− (Load.)	*NA*		*NA*		*NA*	
WLSMV	*NA*	− (Load.)	*NA*		*NA*		*NA*		*NA*	*NA*
Correlated factors^a^	*NA*	+	*NA*	+	*NA*	+	*NA*	+	*NA*	+
Misspecification proportion^a^×										
DWLS	*NA*	−	*NA*	−	*NA*		*NA*		*NA*	
Correlated factors^a^	*NA*	+	*NA*	+	*NA*	+	*NA*	+	*NA*	+
MLR ×										
Asymmetric	+		+	− (Dim.)			+ (1F)		*NA*	*NA*
DWLS ×										
Number of indicators	+									
Asymmetric	+		+				+ (1F)			
Loading magnitude	+		+							
Correlated factors^a^	+	+	+	+	+	+	+	+	+	
WLSMV ×										
Asymmetric	+		+				+ (1F)		*NA*	*NA*
Correlated factors^a^						+			*NA*	*NA*
Loading magnitude ×										
Correlated factors^a^						−				

Summary of main effects and relevant (two-way) interaction effects, taken from Table A3 for correctly specified models and Table A4 for misspecified models (see Additional File 4 of the Supplementary Online Material). Correct = Correctly specified models; Misspecified = Misspecified models; 1F = One-factor CFA; 2F = Two-factor CFA; Dim. = Misspecified factor dimensionality; Load. = Unmodeled cross-loadings. The multiplication sign (×) indicates interaction terms. SRMR is only available for comparing ML and DWLS (because SRMR is identical for ML and MLR, as well as DWLS and WLSMV, Maydeu-Olivares et al., 2018). Lower values of (recoded) GOFs represent worse fit (i.e., χ^2^, χ^2^/*df*, RMSEA, and SRMR multiplied by −1), so “+” means improving fit and “−” means worsening fit with increasing values of the predictor. Blank cells = no substantive effect. Parentheses = effects apply to a subset of scenarios; different effects are separated with a slash (1F/2F and Dim./Load., respectively). *NA* = not applicable (i.e., scenario not tested or testable). ^a^Only for GOFs from two-factor models (2F) and models with unmodeled cross-loadings (Load.)

#### Correctly specified models

Several columns in Table 2 summarize the findings for correctly specified one- or two-factor models. Table 2 only presents relatively large effects from the regression analysis (i.e., relatively large unstandardized regression coefficients). After discussing large main effects, we move on to discussing large interaction effects that only multivariate analysis can uncover. Finally, we report *R*^2^, the total variance of GOFs explained by including all data and analysis characteristics and their two-way interaction effects.

Multivariate regression showed that GOFs were surprisingly susceptible to various characteristics even in correctly specified models. As expected, SRMR depended on the sample size and suggested a better fit with increasing sample size. Likewise, χ^2^ depended on the number of indicators. It suggested better fit with decreasing number of indicators. χ^2^, χ^2^/*df*, and RMSEA (the latter especially in scenarios with one-factor models) suggested better fit for symmetric instead of asymmetric response distributions. The type of estimator impacted all GOFs. Effects were mixed for different GOFs, as confirmed by multivariate regression. Whereas χ^2^, χ^2^/*df*, and RMSEA (the latter in scenarios with one-factor models) indicated better fit when using DWLS instead of ML, CFI and SRMR (the latter especially in scenarios with two-factor models) pointed to worse fit with DWLS.

The type of estimator moderated several effects on GOFs. The number-of-indicator dependency of χ^2^ weakened when switching from ML to DWLS. Likewise, when using MLR, DWLS, or WLSMV instead of ML, the effect of the distribution shape (with varying skewness) vanished. With DWLS instead of ML, increasing loading magnitudes suggested better fit according to χ^2^ and χ^2^/*df*, but not the other GOFs. DWLS also indicated better model fit for correlated than for uncorrelated factors according to all GOFs we tested.

The explained variance (*R*^2^) in the multivariate regression quantifies the joint explanatory power of all simulated characteristics on GOFs, which should ideally be low (as GOFs are otherwise systematically susceptible to these characteristics). For all GOFs, *R*^2^ was consistently higher for correctly specified one-factor than two-factor models (see Table A3 in Additional File 4 of the Supplementary Online Material). The largest shares of explained variance emerged for χ^2^ and SRMR of correctly specified one- and two-factor models (.815 ≤ *R*^2^ ≤ .894), meaning that χ^2^ and SRMR most strongly varied as a function of the simulation characteristics. By comparison, all tested GOFs derived from χ^2^ (i.e., χ^2^/*df*, CFI, and RMSEA) were less influenced by data- and analysis-specific characteristics than χ^2^ (or SRMR, for that matter), which in turn limited the GOF variability for correctly specified models that those characteristics might have explained (.061 ≤ *R*^2^ ≤ .266).

#### Misspecified models

Every second Table 2 column summarizes relevant main and interaction effects for models with misspecified factor dimensionality or unmodeled cross-loadings. We identified those effects as relatively large (or relevant) that were equal to or larger than the main effects of the magnitude or, if applicable, the proportion of misspecification (i.e., relatively large unstandardized regression coefficients). After describing the sensitivity of GOFs to the magnitude or proportion of misspecification, we turn to interactions between misspecification and other characteristics. Then, we explore the susceptibility of GOFs to data and analysis characteristics. Finally, we analyze the explained variance (*R*^2^) of GOFs taking all intended influences (i.e., magnitude and proportion of misspecification) and those of other characteristics together.

Multivariate analysis confirmed that GOFs were sensitive to the magnitude of misspecification. GOFs indicated worse fit as the magnitude of the misspecification increased (i.e., misspecification in factor dimensionality, higher unmodeled cross-loadings). Likewise, increasing the proportion of cross-loadings in the population model but leaving them unmodeled in the analysis model suggested decreasing model fit.

Crucially, the expected sensitivity of GOFs to misspecification varied depending on several other characteristics—a problem that only multivariate analysis could uncover. This *differential* sensitivity of GOFs became evident through substantial two-way interaction effects of the magnitude and proportion of misspecification with the factor correlation (for all GOFs) and the type of estimator (for χ^2^ and χ^2^/*df* only) in scenarios with unmodeled cross-loadings. We specifically draw the reader’s attention to the interaction between the proportion of misspecification and the factor correlation—an interaction already evident in the GOF distributions in Fig. 4 and resurfacing in the multivariate analysis summarized in Table 2. GOFs correctly suggested worse fit with a higher proportion of unmodeled cross-loadings when factors were uncorrelated. When factors were correlated, GOFs somewhat paradoxically suggested better fit. Thus, the factor correlation (i.e., uncorrelated or correlated) moderated the effect of the proportion of unmodeled cross-loadings on GOFs.

With regard to GOFs’ susceptibility to data and analysis characteristics, several findings from the multivariate regression are noteworthy. As the loading magnitude increased, most GOFs typically indicated worse fit (i.e., χ^2^, χ^2^/*df*, RMSEA, and SRMR; the latter especially in scenarios with misspecified factor dimensionality). Only CFI showed a different pattern: It pointed to better model fit with increasing loading magnitudes in scenarios with unmodeled cross-loadings—an effect that vanished with correlated instead of uncorrelated factors. We also observed typical influences of the type of factor correlation (in scenarios with unmodeled cross-loadings) and the type of estimator on all GOFs (in scenarios with either misspecified factor dimensionality or unmodeled cross-loadings). Most GOFs were not simply susceptible to the type of estimator, but differentially so depending on correlating factors (for χ^2^, χ^2^/*df,* CFI, and RMSEA in scenarios with unmodeled cross-loadings). This, too, was a complex interaction that only multivariate analysis could uncover. We return to this interaction when visualizing selected effects.

The magnitude and proportion of misspecification and all other characteristics together explained up to 96% of the variation in GOFs (usually more than 62% in most scenarios; see Table A4 in the Additional File 4 of the Supplementary Online Material). As an exception to this rule, χ^2^ and χ^2^/*df* were not explained (*R*^2^ = .002 at most) in scenarios with misspecified factor dimensionality, so the *R*^2^ pattern speaks favorably of χ^2^ and the χ^2^/*df* ratio as being immune to *systematic* influences of *data and analysis characteristics* but also, and problematically so, as being insensitive to *model misspecification* (at least in our extensive simulation)*.*

### Sensitivity and susceptibility of GOFs: Selected effects visualized

Finally, we visualized selected main and interaction effects that turned out to be substantial for all GOFs. The multivariate regression confirmed a substantial susceptibility of all GOFs to different types of estimators, especially for misspecified models. However, the sensitivity of GOFs to misspecification (i.e., unmodeled cross-loadings) and their susceptibility to the type of estimator were moderated by the type of factor correlation (Table 2). Visualizing these effects highlights the complex dependency of GOFs on these characteristics and the way they interact.

**Fig. 5:**
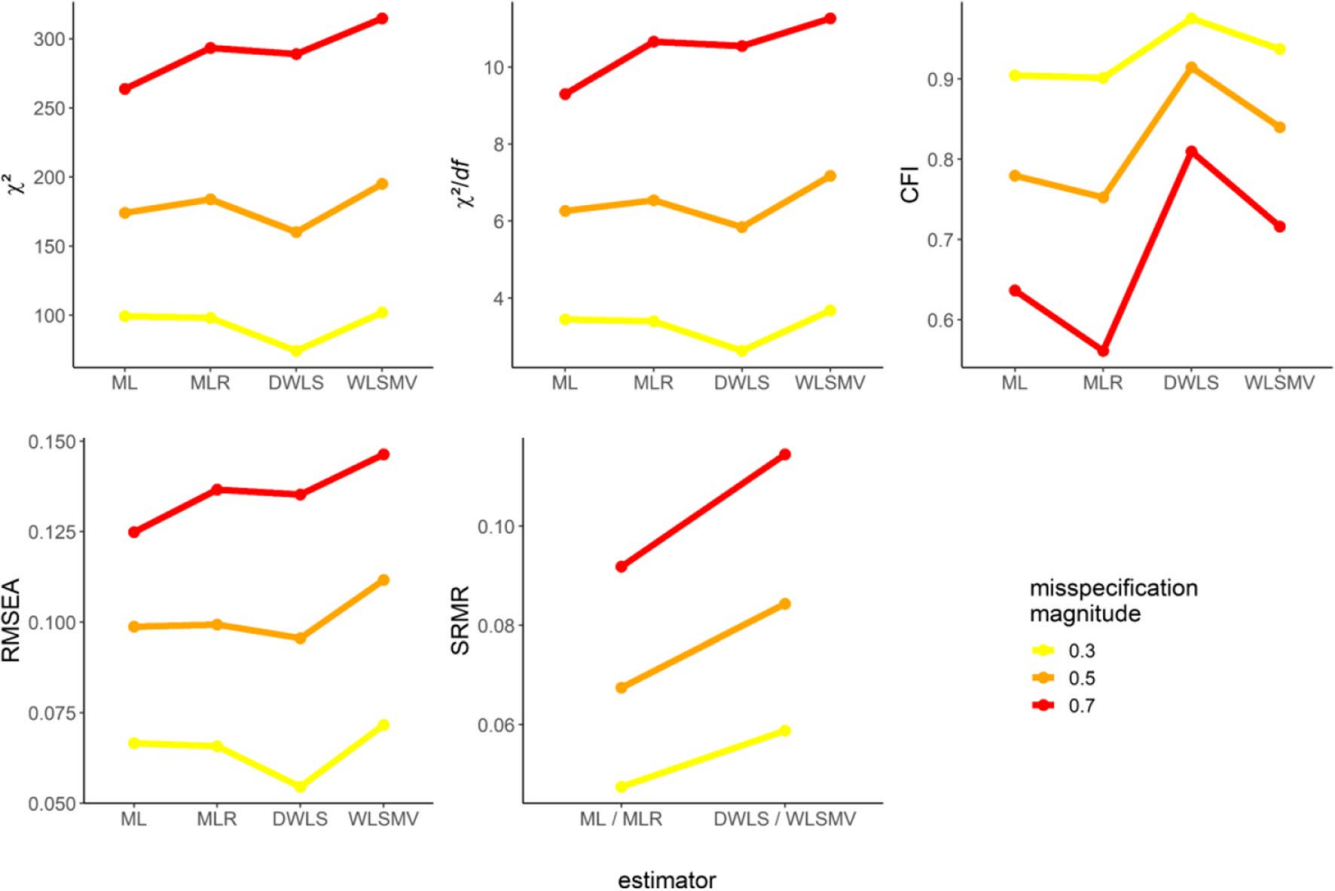
Median values of GOFs conditioned on the type of estimator and misspecification for scenarios with misspecified factor dimensionality. *Note*. GOFs in their original metric and direction

**Fig. 6:**
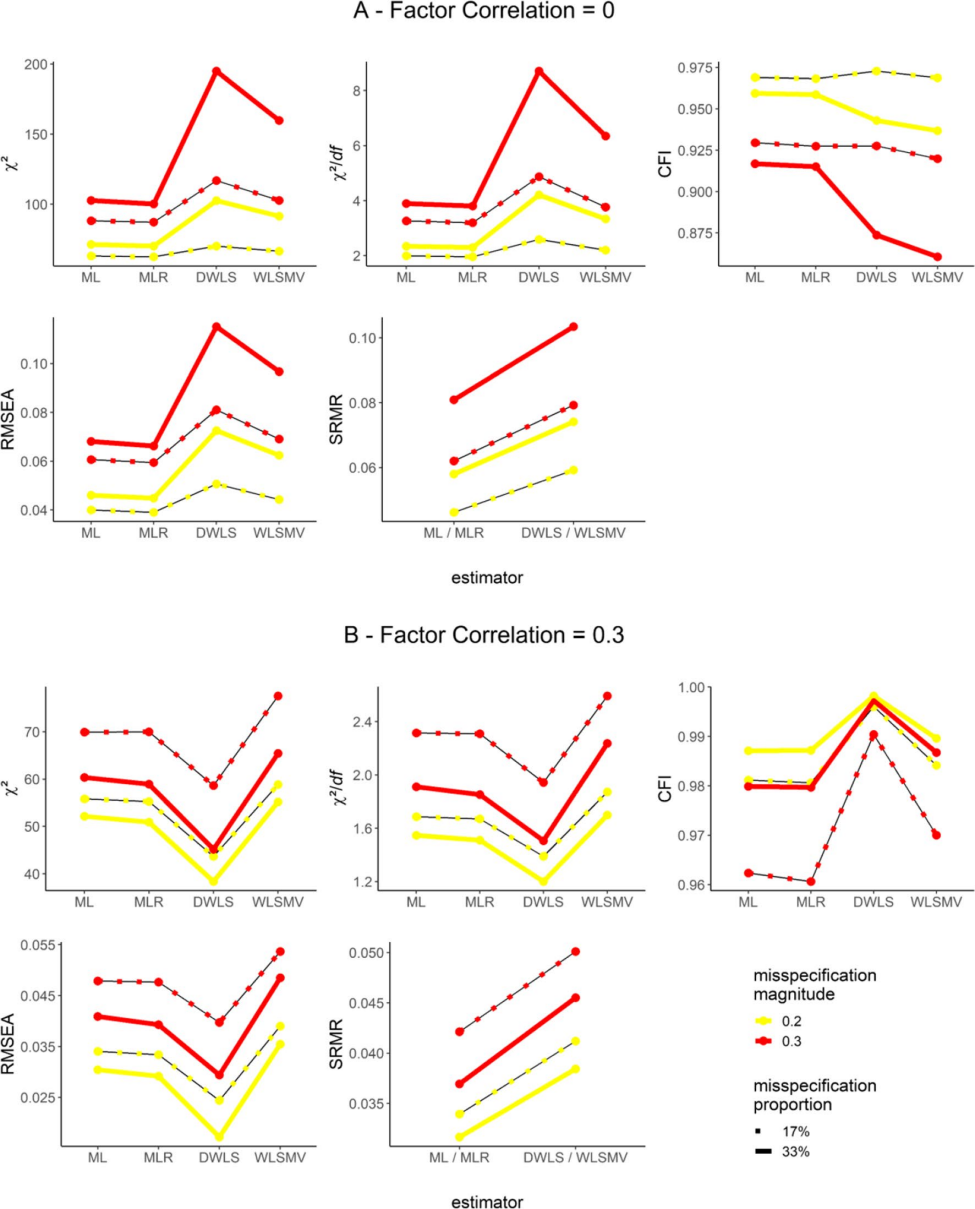
Median values of GOFs conditioned on the type of estimator and misspecification for scenarios with unmodeled cross-loadings**.** *Note*. GOFs in their original metric and direction

Figures 5 and 6 display these interactions via conditional median plots. The *Y*-axis shows the respective GOF and its values (original metric without altering the direction); the *X*-axis conveys the estimators. We disentangled the magnitude and, if applicable, proportion of misspecification by using differentially colored and, if applicable, shaped lines that connect medians for each scenario in the plot. We further split the figures by factor correlation for scenarios with unmodeled cross-loadings.

As a general trend, GOFs were sensitive to misspecification. They correctly indicated worse fit with increasing magnitudes of misspecification across all estimators (Figs. 5 and 6). As expected, a higher proportion of unmodeled cross-loadings also went along with worse fit when factors were uncorrelated. By contrast, a higher proportion of unmodeled cross-loadings suggested better fit when factors were correlated (Fig. 6; compare this to Fig. 4; see also Discussion).

Next, we take a closer look at the susceptibility of GOFs to the type of estimator. A predominant trend was that GOFs were least sensitive to misspecification with DWLS compared to any other estimator (Figs. 5 and 6), except for SRMR. However, the factor correlation moderated this trend. It is capable of being completely reversed. In the presence of uncorrelated factors, GOFs (i.e., χ^2^, χ^2^/*df*, and RMSEA) suggested worse model fit with DWLS than with other estimators (the only exception being CFI when using WLSMV; see Fig. 6).

## Discussion

GOFs were designed to detect model misspecification and help judge the tenability of latent variable models (e.g., Hu & Bentler, 1999). But how well do GOFs fulfill this purpose? We approached this question by conducting the largest and most inclusive simulation study to date on the *sensitivity* of GOFs to model misspecification in CFA models and their *susceptibility* to other data and analysis characteristics. Through this simulation, we were able to integrate, replicate, and extend previous findings on the sensitivity and susceptibility of GOFs. Crucially, data and analysis characteristics other than misspecification should *not* influence GOFs, and the sensitivity of GOFs should not vary depending on such characteristics, lest judgments of model fit may become seriously biased. As we highlight in the following, our simulation results reinforce prior concerns that GOFs clearly fall short of these requirements. This suggests that judging model fit against fixed cutoffs for GOFs—without paying heed to the specific scenario at hand—is a highly problematic practice that researchers should abandon.

Five main insights emerged from our analysis of about 6 × 10^6^ simulated datasets. First, unsurprisingly, GOFs were sensitive to misspecification of both factor dimensionality and cross-loadings: All GOFs correctly indicated worse fit as the degree of misspecified factor dimensionality increased (i.e., the correlation between two factors that were incorrectly modeled as one factor decreased). GOFs also correctly indicated worse model fit as the magnitude and proportion of unmodeled cross-loadings grew (but only when the factors in the model were uncorrelated).

Second, however, the sensitivity of GOFs to model misspecification was not the same across all scenarios. Instead, sensitivity varied considerably depending on several other data and analysis characteristics, especially the type of estimator and the factor correlation in the population model. An intriguing finding was that, when factors were correlated (rather than uncorrelated) in the population and analysis models, GOFs suggested better (rather than worse) model fit as the proportion of unmodeled cross-loadings grew. It may surprise readers that the ability of GOFs to detect misspecification would depend so strongly on the correlation of factors. In hindsight, this finding is plausible: Fitting a correlated two-factor analysis model that ignores substantial cross-loadings in the population model implies a different meaning and orientation of the two factors in the variable space: The factor whose indicators’ cross-loadings go unmodeled reflects a blend of both factors, such that the factor correlation increases. Concomitantly, the estimated factor loadings of indicators with unmodeled cross-loadings are higher than those of correctly modeled indicators (and—by our simulation design—residual variances decrease when cross-loadings are added to the population model). Therefore, a model with correlated factors and substantial cross-loadings that go unmodeled (i.e., are assumed to be zero) accounts for the unmodeled cross-loadings through other model parameters (i.e., the factor correlation and factor loadings), resulting in seemingly good model fit despite clear misspecification. A strong association between the estimated factor correlations and the proportion of unmodeled cross-loadings corroborated this interpretation (tau-b = .54). Put differently, the estimated factor correlations became higher than the induced one (i.e., factor correlation of .30 in the population model). They increased when the proportion of unmodeled cross-loadings increased (0%, 17%, 33% unmodeled cross-loadings: median of estimated factor correlations = .30, .46, .54, respectively). This finding shows that GOFs can be deceptive in certain scenarios, a finding particularly serious in empirical settings in which—contrary to simulation scenarios—the population model remains unknown.

Third, GOFs showed considerable susceptibility to data and analysis characteristics of correctly specified and misspecified models. *All* GOFs analyzed here were susceptible to influences other than model misspecification (especially influences of the type of estimator and factor correlation). The susceptibility of GOFs to data and analysis characteristics differed between correctly specified models, misspecified models, and different kinds of misspecified models. We could replicate several findings of the susceptibility of GOFs to data and analysis characteristics that had been identified previously. Similar to previous studies, we identified a strong dependency of GOFs on the type of distribution (Reußner, 2019) and the type of estimator (Beauducel & Herzberg, 2006; Nye & Drasgow, 2011) in correctly specified models. Like previous studies, we also identified a strong dependency of GOFs on the magnitude of factor loadings (e.g., Beierl et al., 2018; Hancock & Mueller, 2011; Heene et al., 2011) and the type of factor correlation (only with unmodeled cross-loadings; Beauducel & Wittmann, 2005) in misspecified models.

Fourth, we also shed new light on former findings and unraveled hidden complexities of GOFs’ susceptibility to data and analysis characteristics. Most interestingly, former studies (Xia & Yang, 2019) found that DWLS-based GOFs (i.e., χ^2^, CFI, and RMSEA) signaled better fit for misspecified models than ML-based GOFs did. Our results extended this finding. Furthermore, they revealed an interaction with the factor correlation when cross-loadings went unmodeled: DWLS-based GOFs pointed to better fit than ML-based ones with correlated factors; uncorrelated factors reversed the effect.

Fifth, some known influences on GOFs were not as substantial as previously assumed when jointly considering multiple characteristics in a multivariate analysis. For instance, Xia and Yang (2018) found that asymmetric response distributions led to more optimistic model fit evaluations for DWLS-/WLSMV-based GOFs (i.e., χ^2^, χ^2^/*df*, CFI, and RMSEA) for misspecified models than symmetric ones. The same applies to ML-based GOFs (i.e., CFI, RMSEA, and SRMR), as Reußner (2019) found. Though we replicated these principal findings, our main effects of asymmetry, as well as the interaction effects between DLWS/WLSMV and asymmetry, were small relative to other effects in our multivariate analysis. Likewise, the sample size dependency of GOFs (except for SRMR in correctly specified models) remained relatively small compared to other influences in the multivariate analysis—a finding that diverged from what previous studies suggested (e.g., Kenny et al., 2015; Sharma et al., 2005; Shi et al., 2019). These findings highlight the importance of considering the interdependencies among the different influences on GOFs to fully understand the differential sensitivity and susceptibility to extraneous influences on GOFs.

As outlined throughout the paper, we investigated the sensitivity and susceptibility of GOFs for many combinations of characteristics and types of misspecification, extending the scope of previous simulation studies considerably. Still, our enlarged simulations could not cover all (potentially relevant) data and analysis characteristics or types of misspecification. A limitation to be aware of is that we restricted our simulations to CFA models despite the presence of several other models in the structural equation modeling context (see Garrido et al., 2016, for an extensive simulation about fit in exploratory structural equation models) and beyond. Further, we limited ourselves to two types of misspecification (i.e., misspecification due to factor dimensionality and misspecification due to unmodeled cross-loadings), being fully aware that other types of misspecification regularly occur in empirical settings (such as unmodeled residual covariances; see Podsakoff et al., 2003). Such different types of misspecification are likely to impact GOFs differently (e.g., Savalei, 2012; Shi et al., 2018b, 2019; Shi & Maydeu-Olivares, 2020). While covering many scenarios, we certainly do not cover all scenarios one may encounter in research. For example, psychological inventories often require CFA models with more than two factors and more than 12 indicators; to illustrate, the Big Five Inventory–2 (Soto & John, 2017) has 15 factors of facet traits nested in five domain factors and based on 60 indicators in total. Likewise, sample sizes larger than 2000 regularly occur in large-scale assessments (e.g., Programme for the International Assessment of Adult Competencies, PIAAC, has a per-country sample size of at least 4500; OECD, 2013).

## Implications

We acknowledge that the sheer number of results from our simulation can be daunting. However, together these results convey a clear and straightforward message: The *sensitivity of GOFs to model misspecification* varies greatly across simulation scenarios. Moreover, *GOFs are susceptible to various data and analysis characteristics*. GOF values reflect characteristics other than the magnitude and proportion of model misspecification. These conclusions align with those of several other studies as our extensive simulation study replicated several known influences on GOFs (such as their dependency on the type of estimator, e.g., Beauducel & Herzberg, 2006; Xia & Yang, 2019). In addition, we extended and refined the current knowledge on the sensitivity and susceptibility of GOFs by uncovering several relevant moderators through large interaction effects (especially interactions of several characteristics with the type of estimator or the factor correlation). Our findings underline even more strongly than previous findings that GOFs respond to various data and analysis characteristics in complex and hard-to-predict ways.

Therefore, one must not blindly trust the values of GOFs to exclusively reflect (mis)fit, let alone rigidly apply fixed cutoffs for model evaluation. We believe this important insight should be internalized by all researchers who use CFA models, and it should be included in statistics and methods curricula dealing with model evaluation. Moreover, we understand that the findings may sound pessimistic and leave some readers wondering how to approach model evaluation in the future. However, all fundamental issues with GOFs that we and others identified (e.g., Marsh et al., 2004; McNeish & Wolf, 2023a) have a silver lining. They encourage researchers to think more deeply about the appropriateness of fixed cutoffs for GOFs and explore alternative procedures that will ultimately lead to more accurate judgments about whether a model can be accepted.

Below, we first expand on the problem with fixed cutoffs for GOFs that springs from the susceptibility of GOFs to various data and analysis characteristics. Following this, we outline several promising avenues for model evaluations that do not rely on problematic fixed cutoffs.

## (Fixed cutoffs for) GOFs are more problematic than commonly assumed

Considering the findings of our simulation, how solid as a basis for evaluating model fit are fixed cutoffs for GOFs? Our results imply that relying on the same fixed cutoffs to judge model fit in real data applications can be highly problematic and misleading in many settings. Thanks to the breadth of scenarios we studied, we can further illustrate and quantify this problem. To do so, we estimated the frequency distribution of GOFs for correctly specified models separately for each simulation scenario. The 95% quantile (for χ^2^, χ^2^/*df*, RMSEA, and SRMR; 5% quantile for CFI) of each frequency distribution corresponds to a 5% probability of concluding that a model is misspecified when it is, in fact, correctly specified (i.e., 5% Type I error rate). We can use those quantiles as cutoffs for GOFs. Additional File 5 of the Supplementary Online Material (Tables A6–A10) shows the tabulated quantiles.

Researchers often take CFI values above .950 to indicate good model fit (Hu & Bentler, 1999). This heuristic might be sufficiently accurate under some but certainly not under all circumstances. Low loading magnitudes in particular undermine the nominal Type I error rate when using a cutoff of CFI > .950. In some scenarios, *much more lenient* values than .950 maintain a 5% error rate. For example, a cutoff as low as CFI = .813 is fully appropriate to demarcate correctly specified and misspecified models for a one-factor model estimated with ML at a sample size of *N* = 200, with loadings of .40 for six indicators and seven response options, in the presence of asymmetric data. In other scenarios, such as in the presence of high loadings, maintaining a 5% error rate requires *much stricter* values than .950 (e.g., a cutoff of .979 results with loadings of .80 in an otherwise identical scenario). To be very clear, accepting (or rejecting) models under various scenarios at a fixed cutoff (.950) does not effectively control the Type I error rate. Fixed cutoffs cannot do justice to every possible scenario. Consequently, we strongly discourage researchers from inferring the tenability of a model based on conventional, fixed cutoffs.

These examples highlight two caveats about fixed cutoffs, such as those suggested by Hu and Bentler (1999), that have guided researchers’ model evaluations for over two decades. Using cutoffs under scenarios not covered in the initial simulation studies is highly problematic. This pertains, for instance, to testing models with low versus high factor loadings. For model evaluations through GOFs to be valid, researchers need to consider their specific data and analysis characteristics. In this regard, our findings reinforce previous warnings against overgeneralizing cutoffs, including those that Hu and Bentler (1999) stated themselves in their original publication suggesting the canonical cutoffs (see also Marsh et al., 2004; McNeish & Wolf, 2023a; Nye & Drasgow, 2011).

## Moving from fixed to tailored cutoffs is the way forward

Where does this leave researchers seeking to evaluate their model’s fit? We recommend that researchers take three steps. First, researchers should consider and test alternative models to learn more about potentially better-suited models. Second, they should inspect local (mis)fit, for instance, via the residual matrix and modification indices, to investigate whether a model is probably correctly specified or misspecified (see Pornprasertmanit, 2014, for a sophisticated strategy to evaluate local fit). Third, and most promisingly, researchers should inspect global fit not via fixed but via *tailored* (also called “dynamic”; McNeish & Wolf, 2023a, b) cutoffs for GOFs to evaluate the overall model fit free from bias, including any entailed misfit. Whereas considering alternative models and inspecting local fit are time-honored strategies, tailored cutoffs are a much more recent approach that, we believe, holds great promise and offers a much-needed remedy for the issues with GOFs identified in our present simulation. We believe research needs to move toward tailored cutoffs for GOFs that take into account the specific data and analysis characteristics. However, tailored cutoffs are a recent introduction and not yet widely used. To foster the much-needed move toward tailored cutoffs, we outline the procedures for evaluating models via tailored cutoffs in more detail here. We hope to encourage more researchers to consider this emerging strategy. We also provide practical examples and R code illustrating how tailored cutoffs can be implemented.

Tailoring cutoffs for GOFs to the specific data and analysis characteristics can be achieved in different ways, which we denote as the table-based approach, the equation-based approach, and the scenario-specific simulation-based approach. Ultimately, all these approaches are based on simulations; however, they differ in whether the user relies on previous simulation results (as in the table-based and equation-based approach) or has to simulate data themselves to obtain cutoffs (as in the scenario-specific simulation-based approach).

### Table-based approach

The simplest strategy to tailor cutoffs to the specific scenario at hand is to consider tables from simulation studies with scenario-specific cutoffs, such as Tables A6 to A10 in Additional File 5 of the Supplementary Online Material. These tables contain cutoffs for combinations of data and analysis characteristics. They were created to read out the cutoff that can maintain error rates at the desired level in one’s specific empirical setting (i.e., accounting for the data and analysis characteristics). This strategy is easy to apply and reminiscent of looking up critical values of, say, *z*-scores or *t*-statistics. One merely selects cutoffs for GOFs from the simulation scenario most closely resembling one’s own empirical data and analysis characteristics. For example, for a one-factor model with six indicators, five response options, factor loadings around .60, and a symmetric response distribution estimated with WLSMV in a sample of 200 respondents, one would reject the tested model if the χ^2^/*df* ratio is larger than 1.918, CFI is smaller than .972, RMSEA is larger than .068, or SRMR is larger than .048. However, the table-based approach is somewhat limited: If one’s actual data and analysis characteristics are dissimilar to those of simulation scenarios, cutoffs are not given. The other two strategies to arrive at tailored cutoffs go beyond the relatively simplistic table-based strategy to overcome these limitations.

### Equation-based approach

In the equation-based approach,^8^ regression formulae predict tailored cutoffs (Nye & Drasgow, 2011). Formulae originate from a single simulation study containing information about how data and analysis characteristics influence GOFs. Users plug characteristics of their own empirical setting into the formulae to obtain cutoffs.

To exemplify the equation-based approach, we derived regression formulae for tailored cutoffs based on the results of our present simulation. The procedure was as follows: We took the cutoffs of Tables A6 to A10 in Additional File 5 of the Supplementary Online Material as dependent variables and regressed them on all data and analysis characteristics and their quadratic terms and two-way interactions (separately for each GOF). The data and analysis characteristics, as well as their quadratic terms and two-way interactions, explained a large share of the variation in cutoffs for GOFs (*R*^2^ ≥ .810). We saved the regression coefficients in Table 3. The sum of the regression coefficients times the characteristics (i.e., the regression formula) predicts an appropriate cutoff for each GOF. To arrive at appropriate cutoffs for one’s own empirical problem, one plugs their empirical data and analysis characteristics into the regression formulae using the coefficients from Table 3. We included a user-friendly R script in Additional File 6 of the Supplementary Online Material for this purpose. In principle, the regression formulae allow researchers to derive appropriate cutoffs even if their empirical data and analysis characteristics do not perfectly match the ones from the simulation studies.

**Table 3:** Regression coefficients to derive tailored cutoffs

Independent variables	Dependent variable				
	χ^2a^	χ^2^/*df*	CFI	RMSEA	SRMR
Intercept	−23.94201	3.28519	−0.53129	0.13285	0.05279
	Main effects				
Estimator (Reference ML)					
MLR	6.72418	0.45189	−0.21041	0.01536	*NA*
DWLS	5.84976	−0.68404	0.19662	−0.03062	0.03774
WLSMV	−4.68805	−0.27096	0.06079	−0.00865	*NA*
Number of indicators	11.08965	−0.04753	0.04016	−0.00235	0.00278
Response options	−7.16670	−0.35058	0.12387	−0.00896	−0.00963
Response options^2	0.72250	0.03496	−0.00936	0.00098	0.00084
Asymmetric (Reference symmetric)	−0.27294	0.02331	−0.04904	−0.00024	−0.00115
Loading magnitude	−25.73792	−3.58376	4.12967	−0.08865	0.02653
Loading magnitude^2	20.41717	2.96247	−2.75074	0.05766	−0.09506
Sample size	0.00906	0.45022	2.27580	−0.12606	−0.05619
Sample size^2	1.20211	−0.15723	−0.82698	0.04331	0.01882
Number of factors	−12.26618	−0.19792	−0.32211	−0.00594	0.01323
	Two−way interaction effects				
Estimator					
MLR×Number of indicators	−0.49485	−0.01090	0.00247	−0.00041	*NA*
MLR×Response options	0.17085	0.00216	0.00384	0.00005	*NA*
MLR×Response options^2	−0.02131	−0.00052	−0.00024	−0.00001	*NA*
MLR×Asymmetric	−2.71568	−0.08311	−0.00135	−0.00225	*NA*
MLR×Loading magnitude	−7.76460	−0.99175	0.45378	−0.03246	*NA*
MLR×Loading magnitude^2	−2.61117	0.42949	−0.31994	0.01556	*NA*
MLR×Sample size	−3.93101	−0.26550	0.09707	−0.00768	*NA*
MLR×Sample size^2	1.45709	0.09907	−0.03794	0.00311	*NA*
MLR×Number of factors	2.71283	0.11781	−0.00868	0.00304	*NA*
DWLS×Number of indicators	−2.43747	0.00544	0.00158	−0.00038	−0.00022
DWLS×Response options	−0.39327	−0.02550	−0.00440	−0.00033	−0.00758
DWLS×Response options^2	0.02110	0.00195	0.00034	0.00003	0.00058
DWLS×Asymmetric	−3.01669	−0.09613	0.00452	−0.00244	0.00140
DWLS×Loading magnitude	−41.99689	−1.36226	−0.30944	−0.05998	−0.00280
DWLS×Loading magnitude^2	16.73726	0.48895	0.18350	0.02118	−0.00311
DWLS×Sample size	−2.10846	−0.11430	−0.11629	0.02896	−0.02165
DWLS×Sample size^2	0.75628	0.04419	0.04250	−0.00982	0.00756
DWLS×Number of factors	16.86537	0.64662	−0.01075	0.02281	0.00330
WLSMV×Number of indicators	−0.60239	−0.00413	0.00097	−0.00029	*NA*
WLSMV×Response options	0.63654	0.01440	−0.00270	0.00054	*NA*
WLSMV×Response options^2	−0.05539	−0.00115	0.00022	−0.00004	*NA*
WLSMV×Asymmetric	−2.91980	−0.09484	0.00368	−0.00256	*NA*
WLSMV×Loading magnitude	10.29574	0.24493	−0.06415	0.00577	*NA*
WLSMV×Loading magnitude^2	−15.70961	−0.45035	0.03405	−0.01194	*NA*
WLSMV×Sample size	3.01133	0.05021	−0.04314	0.00682	*NA*
WLSMV×Sample size^2	−1.16888	−0.01938	0.01545	−0.00250	*NA*
WLSMV×Number of factors	3.90897	0.15706	−0.00697	0.00452	*NA*
Number of indicators×					
Response options	−0.25997	−0.00776	0.00002	−0.00033	−0.00019
Response options^2	0.02789	0.00081	−0.00003	0.00004	0.00002
Asymmetric	0.17890	0.00040	0.00034	0.00002	0.00003
Loading magnitude	−4.80064	−0.04388	−0.10488	−0.00698	−0.00316
Loading magnitude^2	3.83154	0.04484	0.07017	0.00652	0.00190
Sample size	−1.04664	0.00016	−0.01404	0.00655	−0.00160
Sample size^2	0.38895	0.00157	0.00500	−0.00224	0.00058
Number of factors	0.64889	−0.01164	0.00234	−0.00030	0.00003
Response options×					
Asymmetric	0.47743	0.01356	−0.00319	0.00043	0.00061
Loading magnitude	22.43204	1.53987	−0.33800	0.04504	0.01794
Loading magnitude^2	−19.13312	−1.33866	0.23297	−0.03818	−0.01639
Sample size	2.42094	−0.20111	−0.00951	−0.00561	0.01125
Sample size^2	−1.16974	0.07428	0.00109	0.00208	−0.00388
Number of factors	1.18404	0.04887	0.00555	0.00098	0.00068
Response options^2×					
Asymmetric	−0.04690	−0.00122	0.00031	−0.00005	−0.00006
Loading magnitude	−2.12451	−0.14685	0.02682	−0.00451	−0.00166
Loading magnitude^2	1.79085	0.12730	−0.01863	0.00381	0.00151
Sample size	−0.40475	0.01424	0.00006	0.00030	−0.00097
Sample size^2	0.17973	−0.00524	0.00020	−0.00011	0.00034
Number of factors	−0.10687	−0.00463	−0.00039	−0.00010	−0.00005
Asymmetric×					
Loading magnitude	3.71952	0.27558	0.11280	0.01327	0.00844
Loading magnitude^2	−1.17710	−0.18396	−0.07174	−0.00937	−0.00713
Sample size	0.29781	−0.00253	0.02437	−0.00219	−0.00393
Sample size^2	−0.16484	−0.00346	−0.00870	0.00072	0.00138
Number of factors	−0.81043	−0.03425	−0.00208	−0.00101	−0.00028
Loading magnitude×					
Sample size	16.43214	0.03858	−5.87140	0.01187	−0.04098
Sample size^2	−8.22119	−0.08583	2.14448	−0.00411	0.01586
Number of factors	1.82103	0.21559	0.65988	0.02703	−0.01793
Loading magnitude^2×					
Sample size	−15.03742	−0.14310	3.87122	0.00022	0.06458
Sample size^2	7.54665	0.12608	−1.41187	0.00015	−0.02336
Number of factors	5.02726	0.03351	−0.43878	−0.01413	0.04983
Sample size×					
Number of factors	0.39375	0.08076	0.09529	−0.00988	−0.02326
Sample size^2×					
Number of factors	−0.26943	−0.03848	−0.03378	0.00320	0.00784
Number of factors×					
Correlated factors	−2.51728	−0.05765	0.00487	−0.00223	−0.00481
R^2^	.970	.810	.902	.903	.963
*N*	1296	1296	1296	1296	648

The sum of the regression coefficients times the characteristics (i.e., the regression formula) predicts an appropriate cutoff. Divide the sample size by 1000 before plugging it into the equation. Regression coefficients are unstandardized and uncentered. Independent variables with more than two simulated levels were entered additionally in quadratic form. The multiplication sign (×) indicates interaction terms. SRMR is only available for comparing ML and DWLS (because SRMR is identical for ML and MLR, as well as DWLS and WLSMV, Maydeu-Olivares et al., 2018). *NA* = not applicable (i.e., scenario not tested or testable). ^a^χ^2^ depends on the degrees of freedom and, thus, predicted cutoffs for χ^2^ are barely useful for models different from the ones in the paper.

This approach constitutes a clear advancement over the status quo of rigidly using fixed cutoffs, whatever the preferred heuristic for a GOF is. Further, it is more general than the simplistic table-based approach described first. It is also highly efficient because no new simulation must be carried out (as in the scenario-specific simulation-based approach described next). However, the potential downside is that the starting point is still a single simulation study that can never cover all possible real-world settings, no matter how thorough. Although extrapolation is possible in principle, researchers should only use the regression formulae for tailored cutoffs when empirical settings do not strongly deviate from the simulation scenarios.

### Scenario-specific simulation-based approach

If empirical settings strongly deviate from simulation scenarios, neither cutoff tables nor regression formulae should be used for cutoffs. Instead, one may adopt the third approach and conduct a small-scale, scenario-specific simulation to investigate the behavior of GOFs. Several authors have suggested this approach (most recently, McNeish & Wolf, 2023a, b; for similar earlier work, see Millsap, 2007, 2013; Niemand & Mai, 2018; Pornprasertmanit, 2014; for nested models, see Pornprasertmanit et al., 2013). Before initializing the simulation, researchers define analysis and population models. Then, they simulate data from the population model (via a Monte Carlo simulation, similar to what we did in the present paper), fit the analysis model to the data, and record the GOFs. Similar to our tables in Additional File 5 of the Supplementary Online Material, researchers then extract cutoffs from the resulting GOF distributions. The analysis model can equal (or approximately equal; see Millsap, 2007, 2013; Pornprasertmanit, 2014) the population model, corresponding to a correctly specified model. Cutoffs derived from the GOF distribution of correctly specified models control the Type I error rate (as implemented in the approaches of McNeish & Wolf, 2023a, b; Millsap, 2007, 2013; Niemand & Mai, 2018; Pornprasertmanit, 2014). Including a misspecified model (i.e., where the analysis model differs considerably from the population model) allows one to control the Type II error rate (i.e., the probability of concluding that a model is correctly specified when it is, in fact, misspecified) in the derivation of tailored cutoffs (as implemented in the approaches of McNeish & Wolf, 2023a, b, and Pornprasertmanit, 2014). Further, including several misspecified models might help to evaluate model fit gradually (e.g., McNeish & Wolf, 2023a, b).

Choosing simulation characteristics (e.g., analysis model, sample size, estimator) similar to those of the empirical setting of interest is the gold standard to arrive at tailored cutoffs. By simulating data, cutoffs can be tailored to the setting of interest. However, the flexibility of the scenario-specific simulation-based approach may not always be a merit but also a difficulty. The approach demands specific knowledge about defining population and analysis models, running simulations, and analyzing them. Automated solutions (i.e., Shiny apps) can ease the process considerably (e.g., McNeish & Wolf, 2023a).

In sum, the table-, equation-, and scenario-specific simulation-based approaches are three alternative ways to arrive at tailored cutoffs for model evaluation. Although these procedures are more involved than judging model fit against fixed cutoffs for GOFs, we hope our simulation results have convinced the reader of the urgency of phasing out fixed cutoffs in favor of a more appropriate tailored approach.

## Conclusion

GOFs were designed to detect model misspecification and support the evaluation of model fit. However, our simulation reinforces the view that there are two fundamental problems with GOFs. First, GOFs not only reflect model misspecification but are susceptible to a range of data and analysis characteristics (other than model misspecification). Second, the sensitivity of GOFs to model misspecification also depends on such characteristics. In this regard, a particularly impressive (and alarming) finding was the strong dependence on absolute GOF values and their misspecification sensitivity to the factor correlation and the type of estimator. Such characteristics are irrelevant from the researcher’s point of view for judging model fit or identifying misspecification. Hence, they should ideally have no bearing at all on GOFs. However, our findings converge with—and even expand—previous small-scale simulations suggesting that a range of characteristics other than misspecification influence absolute GOF values.

The pattern of associations between those characteristics and GOFs is complex, as interaction effects attest; it varies for different GOFs and is hard to predict for specific constellations. This complexity means that simple modifications cannot come to the rescue, such as adding or subtracting a constant from cutoff values. The problem lies with fixed cutoffs for GOFs *as such.* Fixed cutoffs cannot do justice to all combinations of data and analysis characteristics researchers encounter in applied settings.

Our findings make it abundantly clear that the conventional practice of relying on fixed cutoffs for GOFs is far more problematic than commonly assumed. Even though previous simulations had raised some of the issues highlighted in our study, the practice has not changed. Hu and Bentler (1999) already cautioned researchers to execute discretion when using their cutoffs (see also McNeish & Wolf, 2023a). However, researchers continue to rely on these cutoffs even in empirical settings markedly different from the simulation scenarios covered by Hu and Bentler (1999) and related studies by Reußner (2019) and Rutkowski and Svetina (2014). For example, fixed cutoffs are often applied to one-factor CFA models, even though Hu and Bentler’s simulations did not include such models (McNeish & Wolf, 2023b). More than 20 years later, our detailed simulation resonates with their initial warnings and brings several additional issues to light. Consequently, we urge researchers to be wary of the problems with fixed cutoffs.

We recommend that researchers routinely adopt the time-honored strategies of inspecting (and reporting) local fit and comparing alternative models instead of relying exclusively on GOFs. Methodologists have long advocated these effective strategies, but these are far from being universally applied in published research. Ultimately, we believe the field needs to move away from relying on fixed cutoffs and toward cutoffs tailored to the specific data and analysis characteristics (e.g., McNeish & Wolf, 2023a, b). Tailored cutoffs offer an appropriate response to the susceptibility of GOFs and the ensuing lack of validity of fixed cutoffs. To contribute to a much-needed shift toward tailored cutoffs, we discussed and developed emerging strategies for implementing tailored cutoffs and pointed to ongoing work that aims to improve these strategies further. We hope our simulation results will encourage researchers to embark on this path, ultimately resulting in valid and replicable research.

## Footnotes

1 Researchers often assume that GOFs can detect all types of misspecification. As Hayduk (2014) demonstrated, χ^2^, which is incorporated in χ^2^/*df*, CFI, and RMSEA, cannot detect any misspecification in certain constellations of population and analysis models. The analysis model may appear to fit perfectly, although a different population model has generated the data. We hereby acknowledge the general notion of close-fitting models that may be seriously misspecified.

2 We term the influences of data and analysis characteristics on GOFs as “problems” or “susceptibilities,” even though many of these are natural (and sometimes even desirable) consequences of the statistical properties of GOFs. In particular, the dependence of χ^2^ on sample size is readily comprehensible. As a strict and formal test, rather than a GOF, χ^2^ depends on the model degrees of freedom. Per definition, the power of χ^2^ to detect model misspecification increases as the sample size grows (e.g., Moshagen & Erdfelder, 2016). From the perspective of researchers, it would be desirable for GOFs to quantify the degree of model misspecification across many data and analysis characteristics, irrespective of other considerations such as sample size or other empirical features. That is, ideally, GOFs should exclusively reflect model misspecification—any other influences on GOFs are undesirable (e.g., Schermelleh-Engel et al., 2003). Therefore, we label any influences on GOFs other than misspecification as “problematic” from a researcher’s perspective.

3 Adding indicators to the model is a common way to vary the model complexity. Beauducel and Herzberg (2006) and Fan and Sivo (2007), for instance, varied the model complexity by changing the number of indicators *and* the number of factors. Moshagen (2012) and Shi et al. (2018a) showed that the number of indicators rather than the number of factors drove the effect of model complexity on resulting model fit.

4 Shi et al. (2018b) only found the effect of model size on SRMR for models with unmodeled cross-loadings but not misspecified factor dimensionality.

5 In particular, Reußner (2019) found that CFI, RMSEA, and SRMR were susceptible to the type of the response distribution when using estimators that assume multivariate normal and continous data (i.e., maximum likelihood, ML). For estimators that make no assumption about the underlying response distribution (i.e., diagonally weighted least squares, DWLS), Xia and Yang (2018) mathematically demonstrated that the number and distribution of response options directly influence GOFs (i.e., χ^2^, χ^2^/*df*, CFI, and RMSEA). Both characteristics determine the precision of polychoric correlations that feature in the fit function of DWLS and the mean and variance adjustment (WLSMV) of the χ^2^ test statistic, which transfers to χ^2^/*df*, CFI, and RMSEA.

6 Savalei (2020) proposed an analytical correction to DWLS-/WLSMV-based GOFs to make them appear like ML-based ones, which has not yet been implemented in major statistical programs like R or Mplus.

7 To obtain ordered categorical indicators and determine the shapes of the resulting response distributions (i.e., symmetric or asymmetric), we cut the initially continuous data by setting different thresholds. To simulate a symmetric distribution of responses, we set thresholds to produce different numbers of equidistant response options: three (thresholds/*z*-values: −0.75, +0.75; corresponding to frequencies of: 23%, 54%, 23%), five (thresholds/*z*-values: −1.20, −0.40, +0.40, +1.20; frequencies: 12%, 23%, 31%, 23%, 12%), or seven (thresholds/*z*-values: −1.25, −0.75, −0.25, +0.25, +0.75, +1.25; frequencies: 11%, 12%, 18%, 20%, 18%, 12%, 11%). To simulate an asymmetric response distribution, we shifted these response options to thresholds/*z*-values of +0.00, +1.04 (frequencies: 50%, 35%, 15%) for the scenario with three response options; −0.39, +0.31, +0.74, +1.28 (frequencies: 35%, 27%, 15%, 13%, 10%) for five response options; and −0.52, +0.00, +0.35, +0.64, +0.99, +1.40 (frequencies: 20%, 20%, 15%, 15%, 10%, 10%, 10%) for seven response options.

8 One can also loosely subsume another approach under the equation-based category: Researchers can derive tailored cutoffs by relying on statistical assumptions of the χ^2^ distribution without and with misspecification (Moshagen & Erdfelder, 2016). Except for the distribution of χ^2^, GOF distributions are unknown. As many GOFs (e.g., RMSEA) incorporate the χ^2^, one can infer their distribution without and with model misspecification from the χ^2^ distribution. A certain quantile of the GOF distribution without misspecification may serve as a cutoff.

## Declarations

### Prior Dissemination

We have published the manuscript as a preprint on PsyArXiv (10.31234/osf.io/5qag3). A previous version of this manuscript has been published in MADOC, an online repository of the University of Mannheim, as part of Katharina Groskurth’s doctoral thesis (https://madoc.bib.uni-mannheim.de/64707/). We confirm that this manuscript—including the simulated data—has not been published elsewhere.

## Abbreviations

CFA: confirmatory factor analysis
CFI: comparative fit index
*df*: degrees of freedom
DWLS: diagonally weighted least squares
GOF: goodness-of-fit index
ML: maximum likelihood
MLR: robust maximum likelihood
RMSEA: root mean square error of approximation
SRMR: standardized root mean square residual
WLSMV: diagonally weighted least squares mean and variance adjusted
